# Real-world analysis of androgen receptor inhibitors in US patients with nonmetastatic castration-resistant prostate cancer: DEAR-EXT study

**DOI:** 10.1038/s41391-026-01099-3

**Published:** 2026-03-18

**Authors:** Neal D. Shore, Daniel J. George, Nasreen Khan, Niculae Constantinovici, Guifang Chen, Mercedeh Ghadessi, Vlasta Hlebec, Alicia K. Morgans

**Affiliations:** 1 START Carolinas/Carolina Urologic Research Center, Myrtle Beach, SC USA; 2 Duke Cancer Institute, Duke University School of Medicine, Durham, NC USA; 3 Bayer HealthCare Pharmaceuticals, Inc., Whippany, NJ USA; 4 Bayer Consumer Care AG, Basel, Switzerland; 5 Dana-Farber Cancer Institute, Boston, MA USA

**Keywords:** Cancer therapy, Prostate cancer

## Abstract

**Background:**

The real-world retrospective DEAR study in patients with nonmetastatic castration-resistant prostate cancer (nmCRPC) demonstrated darolutamide was associated with lower risks of discontinuation and progression to metastatic castration-resistant disease vs enzalutamide and apalutamide. This analysis expands on DEAR, including additional patients and follow-up, comparing prostate-specific antigen (PSA) response, metastasis-free survival (MFS), and overall survival (OS).

**Methods:**

DEAR-EXT was a chart review of electronic medical records from patients in US urology practices who initiated androgen receptor inhibitors (ARIs) for nmCRPC from August 2019–March 2023. Outcomes included time to initial drug discontinuation, time to metastatic castration-resistant prostate cancer (mCRPC), PSA response, MFS, OS, and safety. Adjusted Cox proportional hazards models were used for the primary analysis and inverse probability of treatment weighting and other sensitivity analyses were performed to evaluate the impact of potential selection bias and confounding factors.

**Results:**

Patients (N = 1375) received darolutamide (n = 565, 41%), enzalutamide (n = 609, 44%), or apalutamide (n = 201, 15%). Baseline characteristics were mainly similar across groups. Adjusted risk of discontinuation was significantly lower with darolutamide vs enzalutamide (hazard ratio [HR] 0.73, 95% confidence interval [CI] 0.61–0.88) and vs apalutamide (HR 0.69, 95% CI 0.54–0.89). Adjusted risk of progression to mCRPC was lower with darolutamide vs enzalutamide (HR 0.63, 95% CI 0.50–0.80) and vs apalutamide (HR 0.72, 95% CI 0.53–0.98). MFS was significantly longer for darolutamide vs enzalutamide (HR 0.65, 95% CI 0.53–0.79) and vs apalutamide (HR 0.72, 95% CI 0.55–0.93). The estimated MFS rates at 24/36 months were 72.3%/60.2% with darolutamide, 59.2%/48.3% with enzalutamide, and 63.9%/47.6% with apalutamide. Darolutamide appeared to be associated with higher PSA response, improved survival, and fewer adverse events vs other ARIs in a real-world setting. Results were consistent in a sensitivity analysis.

**Conclusion:**

Darolutamide demonstrated lower discontinuation rates, longer time to progression, and longer MFS vs other ARIs, highlighting its potential to enhance real-world patient outcomes.

**Clinical trial registration:**

ClinicalTrials.gov identifier: NCT06013475.

## Introduction

Nonmetastatic castration-resistant prostate cancer (nmCRPC) is defined by a rising prostate-specific antigen (PSA) level, despite medical or surgical castration, but no visible metastatic disease on conventional imaging [1–3]. An estimated 1–12% of patients with prostate cancer have nmCRPC [4, 5], but a substantial proportion of them are at risk of progression to metastatic castration-resistant prostate cancer (mCRPC). On androgen-deprivation therapy (ADT) alone, at least one-third of patients develop bone metastases within 2 years [6], which is associated with increased morbidity and mortality [7]. Early treatment with effective agents, however, can delay disease progression and prolong patients’ survival. Nonetheless, because this stage of the disease is largely asymptomatic, it is essential to consider treatments that have a manageable adverse event (AE) profile, ensuring they do not adversely affect patients’ quality of life in the long term [3, 6].

In the USA, three androgen receptor inhibitors (ARIs) are approved for the treatment of patients with nmCRPC: darolutamide, enzalutamide, and apalutamide. Darolutamide is a structurally distinct by design and potent ARI that was shown to improve metastasis-free survival (MFS) and overall survival (OS) in the phase III ARAMIS study [8–10]. Enzalutamide [11–13] and apalutamide [14–16] have shown similar benefits in MFS and OS.

In the absence of randomized clinical trials, experience in clinical practice can provide information on real-world effectiveness and tolerability, which is of value in treatment decision-making. This is of particular importance when selecting treatment from available ARIs that have different AE profiles, while aiming to maintain quality of life in patients with relatively asymptomatic nmCRPC. Our prior observational DEAR study (NCT05362149) provided information on real-world utilization and tolerability of ARI treatment, as well as mCRPC progression rates in patients with nmCRPC in US urology practices [17]. Among the 870 patients included in DEAR over a median follow-up of 22–23 months, darolutamide was associated with a lower risk of discontinuation vs enzalutamide (hazard ratio [HR] 0.73, 95% confidence interval [CI] 0.56–0.94) and apalutamide (HR 0.61, 95% CI 0.44–0.85), a lower risk of progression to mCRPC vs enzalutamide (HR 0.59, 95% CI 0.43–0.82) and apalutamide (HR 0.65, 95% CI 0.42–0.99), and a lower frequency of treatment-emergent AEs (TEAEs) vs enzalutamide and apalutamide. The DEAR-EXT study (NCT06013475) expands on DEAR to provide longer-term data based on one further year of patient inclusion and follow-up, and to compare three additional important clinical outcomes: PSA response, MFS, and OS.

## Materials and methods

### Study design and dataset description

DEAR-EXT was a retrospective chart review cohort study (N = 1375) using the Precision Point Specialty Analytics electronic medical record (EMR) prostate cancer database (Supplementary Fig. 1). The study design and detailed patient eligibility criteria have been previously published (see **Supplementary Methods**) [17].

### Outcomes

Endpoints included time from index date (initiation of first ARI for nmCRPC) to discontinuation of the initial ARI, reasons for discontinuation of initial ARI, time from index date to mCRPC progression, PSA response during initial ARI treatment, MFS, OS, and TEAEs. ARI treatment discontinuation was defined as the earliest occurrence of any of the following: evidence of stopping initial ARI treatment (and no restart of the same treatment within 60 days), switch to another ARI, or death. Diagnosis of metastasis was defined as the earliest occurrence of any of the following: presence of International Classification of Diseases (ICD-9 and ICD-10) diagnosis codes (metastasis-lymph nodes [196.*/C77.*]; secondary malignancies [197.*-198.*/C78.*-79,*] or unknown secondary malignancies [199.*/C80,*]), evidence of bone, visceral/soft tissue, or distant nodal metastasis in patient medical records or radiology reports, or drug treatment initiated specifically for mCRPC (i.e., abiraterone acetate, docetaxel, cabazitaxel, sipuleucel-T, mitoxantrone hydrochloride, or radium-223 dichloride). Death information was collected via a combination of EMRs and linked claims data, which derive the death date from health insurance claims, obituary data, and the National Death Index (see **Supplementary Methods** for further details).

### Statistical analyses

Time-to-event outcomes were compared across ARI treatment groups using unadjusted Kaplan–Meier (KM) estimates and adjusted Cox proportional hazards models. See **Supplementary Materials and Methods** for details of statistical methodology [18].

Several sensitivity analyses (e.g., considering death as a competing risk factor for ARI discontinuation and for progression to mCRPC, and an inverse probability of treatment weighting [IPTW] approach to adjust for differences in baseline confounding factors) were conducted to ensure robust findings, and full details are reported in the **Supplementary Materials and Methods**.

## Results

### Patient and baseline characteristics

In total, 1375 patients met the eligibility criteria and were included in the analyses (Supplementary Fig. 2). Of these, 565 (41%) received darolutamide, 609 (44%) received enzalutamide, and 201 (15%) received apalutamide as their first ARI for nmCRPC. During the study, the number of patients with nmCRPC initiating any ARI treatment decreased annually each year from 473 patients in 2020 to 273 in 2022, but the proportion starting darolutamide increased relative to enzalutamide and apalutamide (Supplementary Fig. 3). Patient characteristics were generally similar across the three treatment groups (Table 1).

**Table 1:** Patient characteristics.

Characteristic	Darolutamide (n = 565)	Enzalutamide (n = 609)	Apalutamide (n = 201)	SMD^a^	P-value^b^
Age, median^c^ (IQR), years	80 (73–85)	79 (73–85)	80 (72–85)	0.04	0.75
Race, n (%)				0.08	0.74
White	375 (66.4)	407 (66.8)	141 (70.1)		
Black	116 (20.5)	125 (20.5)	41 (20.4)		
Other^d^	74 (13.1)	77 (12.6)	19 (9.5)		
Insurance, n (%)^e^				0.06	0.34
Commercial	149 (28.2)	146 (24.9)	58 (29.1)		
Public	380 (71.8)	441 (75.1)	141 (70.9)		
Index year, n (%)				0.13	<0.0001
2019 (Aug–Dec)–2020	240 (42.5)	337 (55.3)	102 (50.7)		
2021–2023 (Jan–Mar)	325 (57.5)	272 (44.7)	99 (49.3)		
Gleason score, n (%)				0.21	0.0002
<8	262 (46.4)	223 (36.6)	78 (38.8)		
≥8	211 (37.3)	233 (38.3)	89 (44.3)		
Missing	92 (16.3)	153 (25.1)	34 (16.9)		
Time from CRPC diagnosis, median^c^ (IQR), months	4.7 (1.2–15.4)	3.4 (0.4–15.0)	3.9 (0.4–13.4)	0.06	0.05
PSA category, n (%)^f^				0.07	0.62
<2 ng/mL	217 (39.2)	230 (39.5)	76 (38.2)		
2–10 ng/mL	240 (43.4)	233 (40.0)	82 (41.2)		
>10 ng/mL	96 (17.4)	120 (20.6)	41 (20.6)		
PSADT category, n (%)				0.15	0.07
≤6 months	169 (29.9)	179 (29.4)	59 (29.4)		
>6–10 months	109 (19.3)	102 (16.7)	43 (21.4)		
>10 months	158 (28.0)	142 (23.3)	47 (23.4)		
Missing	129 (22.8)	186 (30.5)	52 (25.9)		
Follow-up time, median^c^ (IQR), months	26.2 (18.0–36.5)	26.2 (15.1–38.6)	25.5 (16.9–38.6)		0.77

*ANOVA* analysis of variance, *CRPC* castration-resistant prostate cancer, *IQR* interquartile range, *PSA* prostate-specific antigen, *PSADT* prostate-specific antigen doubling time, *SMD* standardized mean difference.

^a^SMD values <0.2 were considered a small effect size.

^b^P-values were obtained using chi-square tests for categorical variables, one-way ANOVA for continuous parametric variables, and Wilcoxon–Mann–Whitney tests for skewed continuous variables.

^c^Arithmetically calculated.

^d^Other race includes American Indian or Alaska Native, Asian, Hispanic or Latino, Native Hawaiian or other Pacific Islander, and unknown or unspecified race.

^e^Insurance type was unknown in 36 patients in the darolutamide group, 22 patients in the enzalutamide group, and 2 patients in the apalutamide group.

^f^PSA category was unknown in 12 patients in the darolutamide group, 26 patients in the enzalutamide group, and 2 patients in the apalutamide group.

The median follow-up time was similar in the three treatment groups, at 26.2 months (interquartile range [IQR] 18.0–36.5) in the darolutamide group, 26.2 months (IQR 15.1–38.6) in the enzalutamide group, and 25.5 months (IQR 16.9–38.6) in the apalutamide group (Table 1). The proportion of patients lost to follow-up was also similar: 17 (3.0%) in the darolutamide group, 27 (4.4%) in the enzalutamide group, and 10 (5.0%) in the apalutamide group.

### ARI discontinuation

**Fig. 1:**
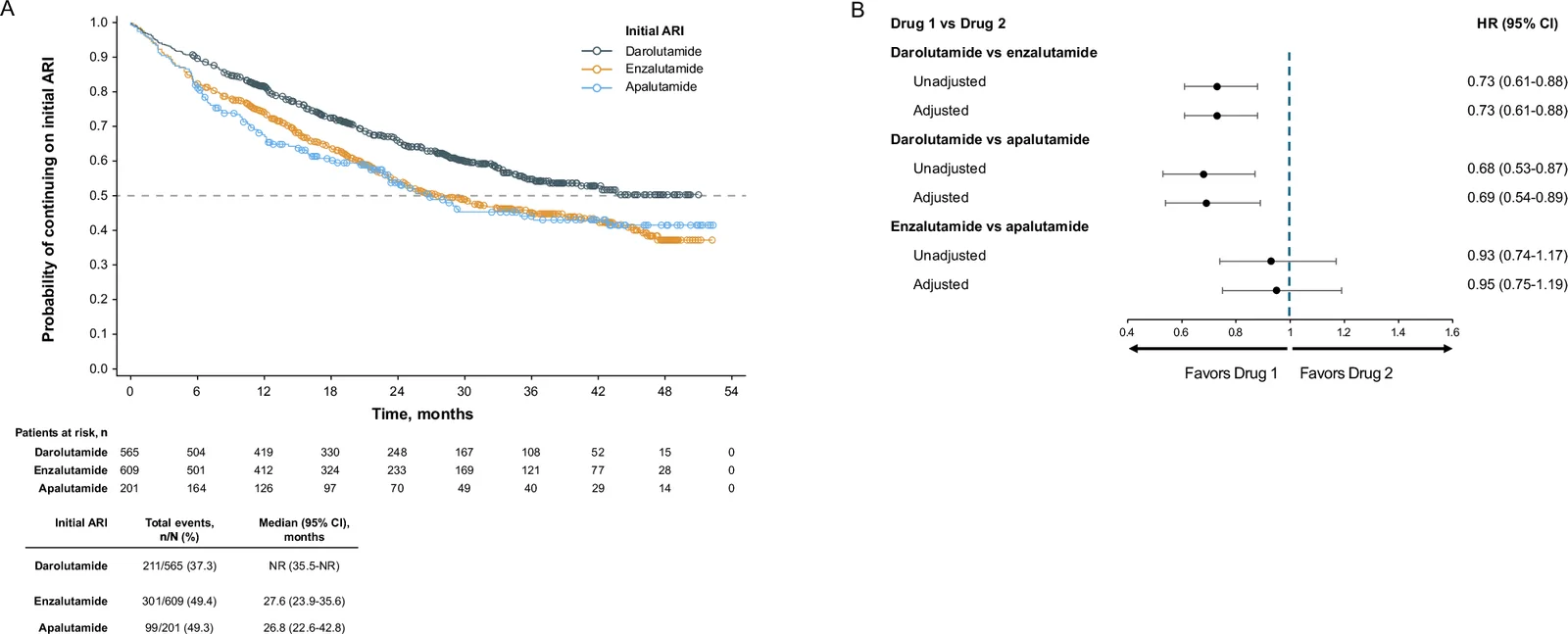
Time to initial ARI discontinuation by treatment group.^a^ **A** Unadjusted Kaplan–Meier estimates and **B** adjusted Cox proportional hazards models. A shows unadjusted Kaplan–Meier estimates and B shows Cox proportional hazards models both unadjusted and adjusted for the following baseline characteristics: age group (≤74, 75–84, ≥85 years), race (White, Black/African American, other/unknown), insurance coverage (commercial, public), index year for ARI initiation (2019–2020, 2021–2023), time (months) from nmCRPC diagnosis to index date, baseline PSA groups (<2, 2–< 10, ≥10 ng/mL), baseline PSA doubling time (≤6, >6–≤ 10, >10 months, missing), and Gleason score at prostate cancer diagnosis (4–7, 8–10, missing). ARI androgen receptor inhibitor, CI confidence interval, HR hazard ratio, nmCRPC nonmetastatic castration-resistant prostate cancer, NR not reached, PSA prostate-specific antigen. ^a^Discontinuation defined as initial ARI treatment stop (and no restart of the same treatment within 60 days), switch to another ARI, or death.

Overall, 611 patients (44.4%) discontinued their initial ARI treatment during the study period: 211 (37.3%) in the darolutamide group, 301 (49.4%) in the enzalutamide group, and 99 (49.3%) in the apalutamide group. The most frequent reasons for discontinuation included AEs, disease progression, and death (Supplementary Fig. 4). The AE most frequently leading to treatment discontinuation was fatigue, reported in 4.2% of patients receiving darolutamide, 6.9% of patients receiving enzalutamide, and 4.0% of patients receiving apalutamide (Supplementary Table 1). Based on the unadjusted KM plots, the median time to ARI discontinuation was not reached in the darolutamide group, was 27.6 months (95% CI 23.9–35.6) in the enzalutamide group, and was 26.8 months (95% CI 22.6–42.8) in the apalutamide group (Fig. 1A). HR estimates from the unadjusted and adjusted Cox proportional hazards model for time to initial ARI discontinuation are provided in Fig. 1B and Supplementary Fig. 5A. The risk of treatment discontinuation was significantly reduced by 27% for darolutamide vs enzalutamide (HR 0.73, 95% CI 0.61–0.88) and by 31% for darolutamide vs apalutamide (HR 0.69, 95% CI 0.54–0.89), whereas the risk of treatment discontinuation was similar for enzalutamide and apalutamide (HR 0.95, 95% CI 0.75–1.19).

### Effectiveness outcomes

**Fig. 2:**
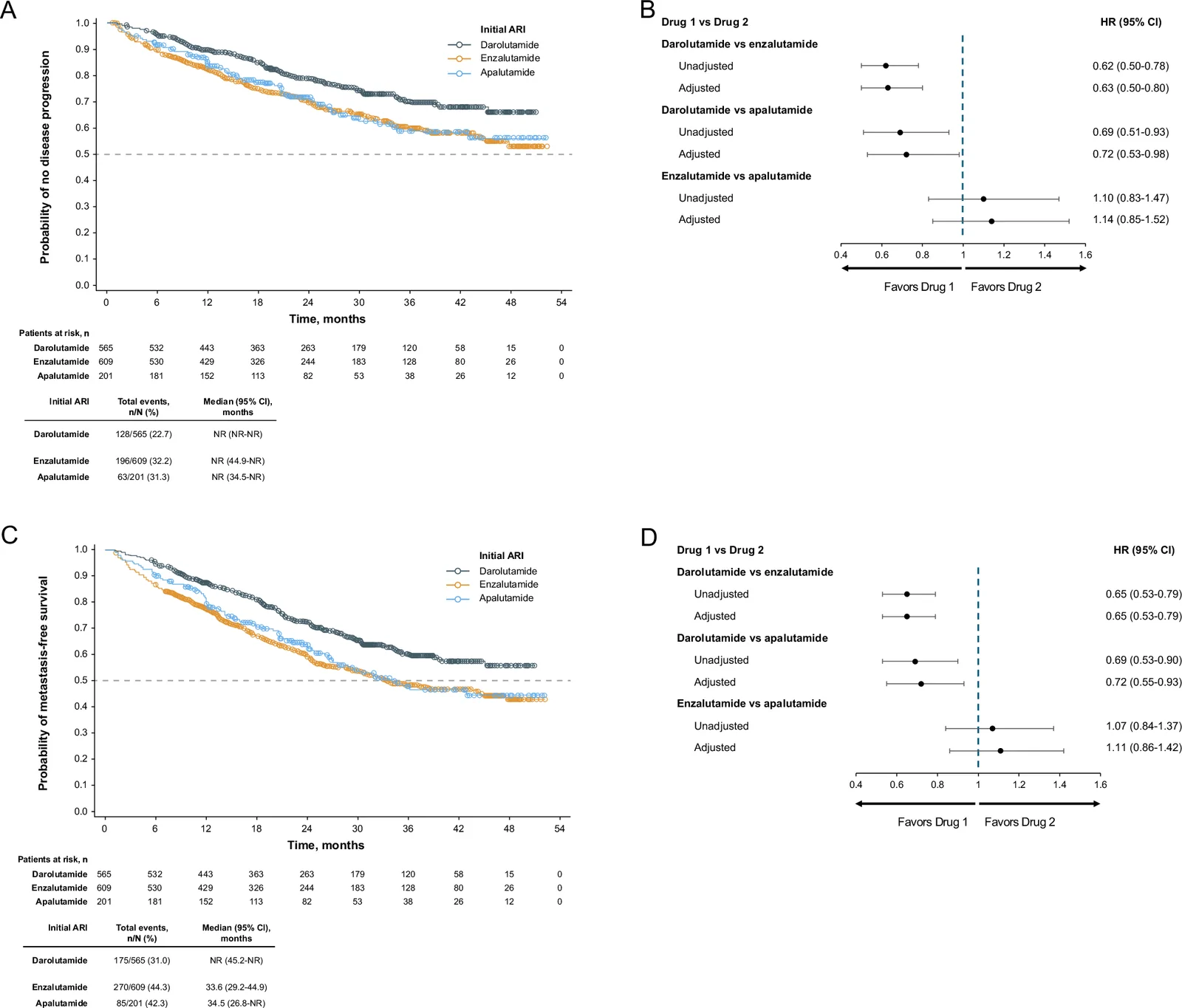
Effectiveness outcomes. Time to mCRPC progression:^a^ **A** Kaplan–Meier estimates and **B** adjusted Cox proportional hazards models; MFS: **C** Kaplan–Meier estimates and **D** adjusted Cox proportional hazards models. A and C show unadjusted Kaplan–Meier estimates and B and D show Cox proportional hazards models both unadjusted and adjusted for the following baseline characteristics: age group (≤74, 75–84, ≥85 years), race (White, Black/African American, other/unknown), insurance coverage (commercial, public), index year for ARI initiation (2019–2020, 2021–2023), time (months) from nmCRPC diagnosis to index date, baseline PSA groups (<2, 2–< 10, ≥10 ng/mL), baseline PSA doubling time (≤ 6, >6–≤ 10, >10 months, missing), and Gleason score at prostate cancer diagnosis (4–7, 8–10, missing). ARI androgen receptor inhibitor, CI confidence interval, HR hazard ratio, mCRPC metastatic castration-resistant prostate cancer, MFS metastasis-free survival, nmCRPC nonmetastatic castration-resistant prostate cancer, NR not reached, PSA prostate-specific antigen. ^a^mCRPC defined by recorded evidence of metastases or use of mCRPC treatment.

Based on unadjusted KM plots, of the 1375 patients, 387 (28.1%) experienced disease progression to mCRPC: 128 (22.7%) in the darolutamide group, 196 (32.2%) in the enzalutamide group, and 63 (31.3%) in the apalutamide group (Fig. 2A). The unadjusted Cox proportional hazards regression model suggested a significantly lower treatment discontinuation risk associated with darolutamide (Fig. 2B). Adjusted HRs demonstrated a significantly lower risk of progression with darolutamide: a 37% lower risk of progression vs enzalutamide (HR 0.63, 95% CI 0.50–0.80) and a 28% lower risk vs apalutamide (HR 0.72, 95% CI 0.53–0.98); no difference in risk of progression was seen between enzalutamide and apalutamide (HR 1.14, 95% CI 0.85–1.52) (Fig. 2B, Supplementary Fig. 5B).

MFS events (metastasis/death) were recorded in 530 (38.5%) of the 1375 patients overall, 175 (31.0%) in the darolutamide group, 270 (44.3%) in the enzalutamide group, and 85 (42.3%) in the apalutamide group (Fig. 2C). The estimated MFS rates at 24 months were 72.3% (95% CI 68.0–76.1) in the darolutamide group, 59.2% (95% CI 54.9–63.3) in the enzalutamide group, and 63.9% (95% CI 56.3–70.5) in the apalutamide group. The estimated MFS rates at 36 months were 60.2% (95% CI 55.0–65.1) in the darolutamide group, 48.3% (95% CI 43.6–52.9) in the enzalutamide group, and 47.6% (95% CI 38.8–55.9) in the apalutamide group. The unadjusted KM median MFS was not reached in the darolutamide group, was 33.6 months (95% CI 29.2–44.9) in the enzalutamide group, and was 34.5 months (95% CI 26.8–not reached) in the apalutamide group. The unadjusted Cox proportional hazards regression model suggested a significantly lower risk of metastasis or death associated with darolutamide (Fig. 2D). Adjusted HRs show the risk of metastasis or death was significantly reduced by 35% with darolutamide vs enzalutamide (HR 0.65, 95% CI 0.53–0.79) and by 28% vs apalutamide (HR 0.72, 95% CI 0.55–0.93), whereas no difference in MFS was observed between enzalutamide and apalutamide (HR 1.11, 95% CI 0.86–1.42) (Fig. 2D, Supplementary Fig. 5C).

Among 951 patients with a PSA level of >1.0 ng/mL at baseline, 802 patients (84.3%) reached ≥50% reduction in PSA from baseline (PSA50), 610 (64.1%) reached ≥90% reduction in PSA from baseline (PSA90), and 489 (51.0%) reached PSA <0.2 ng/mL during the treatment period. For PSA50, the rates were 87.6% for darolutamide, 82.6% for enzalutamide, and 80.0% for apalutamide (Table 2). For PSA90, the rates were 65.2% for darolutamide, 64.3% for enzalutamide, and 60.7% for apalutamide. For PSA <0.2 ng/mL, the rates were 53.2% with darolutamide, 50.6% with enzalutamide, and 48.6% with apalutamide.

**Table 2:** PSA response and OS probability over time.

Outcome		Darolutamide (n = 402)	Enzalutamide (n = 409)	Apalutamide (n = 140)
PSA response,^a^ patients with event, n (%)	PSA50	352 (87.6)	338 (82.6)	112 (80.0)
	PSA90	262 (65.2)	263 (64.3)	85 (60.7)
	PSA <0.2 ng/mL	214 (53.2)	207 (50.6)	68 (48.6)
		(n = 565)	(n = 609)	(n = 201)
OS,^b^ survival probability, % (95% CI)	12 months	95.9 (94–97)	92.0 (90–94)	94.9 (91–97)
	24 months	87.9 (85–91)	78.4 (75–82)	86.0 (80–90)
	36 months	77.6 (73–81)	70.5 (66–74)	74.0 (66–81)
Deaths, n (%)		104 (18.4)	156 (25.6)	47 (23.4)

*ARI* androgen receptor inhibitor, *OS* overall survival, *PSA* prostate-specific antigen, *PSA50* ≥50% reduction in PSA from baseline, *PSA90* ≥90% reduction in PSA from baseline.

^a^PSA response was defined as PSA50, PSA90, or an absolute PSA level of <0.2 ng/mL at any time point during the study. PSA levels <0.2 ng/mL were considered undetectable.

^b^OS was defined as the time from the index date (first ARI received) to the date of death due to any cause. Death information was collected via a combination of electronic medical records and linked claims data, which derive the death date from health insurance claims, obituary data, and the National Death Index.

Overall, 307 of 1375 patients (22.3%) died: 104 (18.4%) in the darolutamide group, 156 (25.6%) in the enzalutamide group, and 47 (23.4%) in the apalutamide group (Table 2). OS rates were numerically higher with darolutamide vs enzalutamide and apalutamide: KM estimates at month 12 demonstrated a 95.9% (95% CI 93.9–97.2) survival with darolutamide, 92.0% (95% CI 89.6–93.9) with enzalutamide, and 94.9% (95% CI 90.8–97.2) with apalutamide. At month 36, OS estimates were 77.6% (95% CI 73.0–81.4) with darolutamide, 70.5% (95% CI 66.2–74.3) with enzalutamide, and 74.0% (95% CI 65.7–80.5) with apalutamide (Table 2).

Supplementary Fig. 6 presents state sequence plots for each treatment group. A lower proportion of patients initiating darolutamide experienced treatment discontinuation (11%) or progression to mCRPC (6%) at 12 months vs those receiving enzalutamide (treatment discontinuation: 13%; progression to mCRPC: 12%) and apalutamide (treatment discontinuation: 22%; progression to mCRPC: 8%).

### Sensitivity analysis

Sensitivity analyses using IPTW to estimate the marginal HRs showed the same trend as the primary analysis results based on adjusted (conditional) HR (see Supplementary Results and Supplementary Figures 7–9). Standardized mean differences of covariates before and after IPTW adjustment are presented for darolutamide vs enzalutamide and darolutamide vs apalutamide in Supplementary Figure 10. Following IPTW adjustment, SMDs were relatively small, suggesting that the covariates were balanced across cohorts.

### TEAEs

TEAEs were reported in 375 patients (27.3%) overall: 25.1% of patients receiving darolutamide, 28.4% of patients receiving enzalutamide, and 29.9% of patients receiving apalutamide (Supplementary Table 2). Overall, the most common TEAE of interest was fatigue, reported in 12.9% of patients, followed by rash, reported in 2.3% of patients. Cognitive and memory impairment, hypertension, and fall were reported in <2% of all patients; no fractures were reported.

## Discussion

For patients with nmCRPC, treatment choice is pivotal, not only to prolong survival but also to minimize the burden of AEs, ensuring adherence to therapy and preservation of quality of life. The ARIs darolutamide, enzalutamide, and apalutamide have demonstrated efficacy and tolerability in phase III clinical trials, showing improvements in MFS and OS compared with ADT monotherapy [8–16]. However, no randomized clinical trials have directly compared these three ARIs, leaving unanswered questions about comparative tolerability and effectiveness. To address this gap, one approach is to use the evidence from phase III clinical trials in a network meta-analysis (NMA). Wenzel et al. (2022) assessed OS and AEs for darolutamide, enzalutamide, and apalutamide using summary data from the pivotal phase III trials. While all three ARIs provided longer OS compared with ADT (HRs: darolutamide 0.69, enzalutamide 0.73, apalutamide 0.79), darolutamide had the highest likelihood of maximum OS benefit and the lowest likelihood of grade ≥3 AEs [19]. Although this is an important insight, indirect comparisons using an NMA may be influenced by study heterogeneity such as differences in populations, and how outcomes are measured. Furthermore, patient-level data are typically not used. Given this, in the absence of head-to-head clinical trials, we sought to perform a real-world comparative study to provide clinically relevant and generalizable results that reflect a broader patient population to provide practical information and inform treatment choices.

Our previous DEAR study demonstrated significantly improved patient outcomes with darolutamide compared with enzalutamide and apalutamide [17]. Darolutamide demonstrated a lower risk of treatment discontinuation vs enzalutamide and apalutamide by 27% (HR 0.73, 95% CI 0.56–0.94) and 39% (HR 0.61, 95% CI 0.44–0.85), respectively, and a lower risk of progression to mCRPC vs enzalutamide and apalutamide by 41% (HR 0.59, 95% CI 0.43–0.82) and 35% (HR 0.65, 95% CI 0.42–0.99) [17], respectively, while maintaining its well-tolerated safety profile reported in clinical trials. While the results were helpful in terms of informing treatment choice, lingering questions remain regarding additional outcomes such as PSA response and MFS, as well as longer-term follow-up and stability of these results in a larger patient population.

DEAR-EXT extends the DEAR study by including 505 more patients (a total of 1375 eligible patients) and an additional year of follow-up, and further endpoints of interest such as PSA response, MFS, and OS. The DEAR-EXT analysis included patients with similar characteristics all with a similar median duration of follow-up (darolutamide: 26.2 months, enzalutamide: 26.2 months, and apalutamide: 25.5 months). In this analysis, darolutamide consistently showed a significantly lower risk of treatment discontinuation after adjusting for baseline characteristics (27% risk reduction vs enzalutamide, and 31% vs apalutamide) and mCRPC progression (37% risk reduction vs enzalutamide, and 28% vs apalutamide). Further, treatment with darolutamide appeared to be associated with longer MFS. At 36 months, estimated MFS rates were 60% with darolutamide, 48% with enzalutamide, and 48% with apalutamide. By 36 months, the median MFS was reached with enzalutamide (34 months) and apalutamide (35 months), but not with darolutamide. The above findings were consistently confirmed in sensitivity analyses. The proportions of patients with nmCRPC receiving apalutamide achieving PSA50 and PSA90 during this study were similar (80% and 61%, respectively) to those reported by 6 months in the phase III SPARTAN study (90% and 57%, respectively [20]). Similarly, in the phase III PROSPER study of enzalutamide in patients with nmCRPC, 76% of patients achieved a confirmed PSA50 [12], similar to that seen (83%) in this real-world population. In a multicenter retrospective study of 191 patients with nmCRPC treated with apalutamide or enzalutamide, patients with PSA50 or PSA90 accounted for 75% and 49% of patients receiving enzalutamide, respectively, and 76% and 43% of patients receiving apalutamide, respectively [21]. The proportions of patients receiving apalutamide or enzalutamide achieving PSA50 and PSA90 were higher in the real-world patient population of the current study but were still numerically lower than seen in patients on darolutamide (PSA50: 88% and PSA90: 65%).

Numerically higher OS probabilities over time were observed with darolutamide in this real-world patient population (estimated OS at 36 months: darolutamide, 78%; enzalutamide, 71%; and apalutamide, 74%), and a numerically lower incidence of TEAEs compared with enzalutamide and apalutamide.

To date, other than DEAR, studies comparing the effectiveness and tolerability of darolutamide, enzalutamide, and apalutamide for nmCRPC in a real-world setting have not been reported. Anecdotal real-world evidence and a few pharmacokinetic studies have suggested that darolutamide has limited potential for clinically relevant drug–drug interactions (DDIs) and thus potential for better tolerability [22], when compared with enzalutamide and apalutamide [23, 24]. In one real-world study of over 1500 men with nmCRPC, only 5% of those treated with darolutamide had potential DDIs, compared with 54% of patients treated with enzalutamide and 58% treated with apalutamide [23]. The lower risk of darolutamide discontinuation seen in this study reflects clinical trial findings showing that it is ranked first among ARIs in terms of the lowest incidence of grade ≥3 AEs; this is of great interest to clinicians when treating patients with nmCRPC [19]. The biological reason for this advantage of darolutamide is considered to be its lower blood–brain barrier penetrative ability relative to other ARIs, together with the aforementioned reduced potential for DDIs [19].

In addition to accurate disease staging based on improved detection of metastases, for example, using prostate-specific membrane antigen positron emission tomography [25], systemic treatment decisions must be based on the benefit–risk profiles of available therapies. The benefits of upfront ARI therapy ahead of chemotherapy have been reported for patients with metastatic disease regardless of tumor burden [26, 27]. Since nmCRPC is a largely asymptomatic stage of the disease, a manageable long-term AE profile is an even more important consideration. Taken together, the differences in efficacy, tolerability, and DDIs of different ARIs have implications for treatment decision-making and potentially for the broader healthcare system. Shore et al. (2022) examined AEs reported in phase III clinical trials and quantified the associated cost using the US Healthcare Cost and Utilization Project database [28]. After adjusting for cross-trial differences, darolutamide was associated with reduced hospitalization costs related to AEs compared with enzalutamide and apalutamide [28].

In general, the interpretation of findings from retrospective, observational studies using data from clinical practice is limited by factors such as potential selection bias, confounding, unmeasured risks, and missingness or measurement errors, which may influence the results. Specifically in this study, unmeasured prescribing bias whereby frail or elderly patients may have preferentially received darolutamide based on its tolerability profile cannot be ruled out. Further limitations of this real-world study may also include the lack of standardization of tests, follow-up information, and variation in follow-up procedures across sites. Moreover, AE reporting is based on physician-reported chart entries, introducing the potential for underreporting. However, a key strength of the study is that a single database with consistent definitions was used across all three cohorts. To address selection bias and confounding factors, adjusted Cox proportional hazards models and several sensitivity analyses were performed, consistently suggesting that darolutamide was tolerable and effective. Considering that the data presented in this study were collected exclusively from community urologist private practices, there may be limitations when generalizing our findings to patients who have been treated by an oncologist or in an academic setting, due to differences in patients’ social or economic characteristics. Nonetheless, we believe that most US patients with nmCRPC are likely treated by community urologists. Moreover, one may argue that the source of data is an important strength of this study, since it encompasses 80% of community urology practices in the USA, with a long follow-up, allowing reasonable comparison of clinically relevant outcomes across the three ARIs approved in the USA.

## Conclusion

In conclusion, data from 1375 patients with >2 years of follow-up suggest that darolutamide may offer favorable real-world outcomes and be a potentially optimal treatment for men with nmCRPC. In the absence of prospective head-to-head trials, this large study provides valuable information to support treatment decision-making, by suggesting both clinical effectiveness and tolerability advantages with darolutamide among patients in a real-world setting. The findings from this study contribute real-world evidence that may help inform future clinical and payer considerations in the management of nmCRPC.

## Supplementary information

Supplementary material

## Data Availability

The data supporting this study’s findings are available from the Precision Point Specialty Analytics electronic medical record prostate cancer database. The data were used under license for the current study and are not publicly available. Interested researchers should contact Specialty Networks (https://specialtynetworks.com).
