# Supplementary material for: Real-world analysis of androgen receptor inhibitors in US patients with nonmetastatic castration-resistant prostate cancer: DEAR-EXT study
Source: Prostate Cancer Prostatic Dis. 2026 Mar 18;29(5):982–9. doi: 10.1038/s41391-026-01099-3 (PMC13719525; doi:10.1038/s41391-026-01099-3)
Supplement: Supplementary file 1 [file 41391_2026_1099_MOESM1_ESM.docx]

# Real-World Analysis of Androgen Receptor Inhibitors in US Patients with Nonmetastatic Castration-Resistant Prostate Cancer: DEAR-EXT Study

**Running head:** ARIs in US nmCRPC patients: DEAR-EXT

Neal D. Shore,^1^ Daniel J. George,^2^ Nasreen Khan,^3^ Niculae Constantinovici,^4^ Guifang Chen,^3^ Mercedeh Ghadessi,^3^ Vlasta Hlebec,^4^ and Alicia K. Morgans^5^

*^1^START Carolinas/Carolina Urologic Research Center, Myrtle Beach, SC, USA;* [*nshore@auclinics.com*](mailto:nshore@auclinics.com)*; ^2^Duke Cancer Institute, Duke University School of Medicine, Durham, NC, USA; ^3^Bayer HealthCare Pharmaceuticals, Inc., Whippany, NJ, USA; ^4^Bayer Consumer Care AG, Basel, Switzerland; ^5^Dana-Farber Cancer Institute, Boston, MA, USA*

**Corresponding author:** Professor Neal D. Shore, START Carolinas/Carolina Urologic Research Center, 823 2nd Parkway, Myrtle Beach, SC 29572, USA. Phone: +1 (843) 347-2450. Email: [nshore@auclinics.com](mailto:nshore@auclinics.com). ORCID ID: 0000-0001-5767-0548.

**Supplementary Materials and Methods**

### Study Design and Dataset Description

In brief, DEAR-EXT was a retrospective chart review cohort study using the Precision Point Specialty Analytics (PPS) electronic medical record (EMR) prostate cancer database (**Supplementary** **Figure 1**). At the time of this study, the PPS network included 80% of US community private-practice urologists in the USA, spanning 2400 locations in more than 1200 cities in 49 states. Patients’ health insurance was covered by Medicare/Medicaid or commercially. Patients treated in academic settings or in oncology practices were not included.

De-identified patient demographic and clinical information was extracted from structured and unstructured data elements (patient charts) recorded as part of routine clinical care in the EMR system. Patient demographics, clinical characteristics, and therapy start dates were primarily obtained from the structured data. These structured data were further enriched during chart review and additional clinical information (eg, treatment stop date, treatment interruption, reasons for discontinuation, dose changes, treatment-emergent adverse events [TEAEs], and AE management) was also recorded. Information on death was collected via a combination of EMR, linked claims data, obituary data, and the National Death Index.

### Patient Eligibility Criteria

Detailed eligibility criteria have been published previously.^17^ Briefly, patients were men aged ≥18 years who initiated treatment with darolutamide, enzalutamide, or apalutamide for the first time for nonmetastatic castration-resistant prostate cancer (nmCRPC) between August 2019 and March 2023 (index date); had evidence of nmCRPC before or within 90 days after the index date; and had at least 6 months of EMR activity after the index date (unless the patient died earlier). Patients were excluded if they had evidence of metastatic disease or a treatment prescribed specifically for metastatic prostate cancer (abiraterone acetate, cabazitaxel, docetaxel, mitoxantrone hydrochloride, radium RA 223 dichloride, or sipuleucel-T) before or up to 30 days after the index date. A history of other primary cancers (except for non-melanoma skin cancer) within 5 years before the index date or inclusion in any clinical trials during the study period were additional exclusion criteria.

### Outcomes

Androgen receptor inhibitor (ARI) discontinuation was defined as earliest occurrence of any of the following: evidence of initial ARI treatment stop (treatment interruption >60 days), switch to another ARI, or death. Reasons for discontinuation of initial ARI were categorized as AE-related, disease progression, death, ARI switch, other, or unknown. Time to metastatic castration-resistant prostate cancer (mCRPC) progression was defined as the time from the index date to the date of first recorded evidence of metastases or use of a treatment specific for metastatic prostate cancer. Prostate-specific antigen (PSA) response was measured only in patients with a PSA level of more than 1.0 ng/mL within 6 months before or at the index date (baseline level). Additionally, patients were excluded from this analysis if they had no PSA measurements at baseline or during the follow-up period. PSA response was defined as a decrease of at least 50% from the baseline PSA level, a decrease of at least 90% from the baseline level, or an absolute PSA level of less than 0.2 ng/mL at any time point during the index treatment period. Metastasis-free survival (MFS) was defined as the time from the index date to the date of first recorded evidence of metastasis or death from any cause. Overall survival was defined as the time from the index date to the date of death due to any cause. TEAEs were recorded as the frequency of all AEs, AEs of special interest (fatigue, rash, cognitive and memory impairment, falls, fractures, and hypertension), and AEs during ARI treatment or 30 days after discontinuation.

### Statistical Analyses

Quantitative continuous or discrete variables were described using medians and interquartile ranges. Qualitative or categorical variables were described by number count and percentage of patients in each category. Standardized mean differences (SMDs) measure the difference between treatment groups while accounting for data spread. SMDs <0.2 were considered to indicate a small difference between groups. Time-to-event outcomes were compared using unadjusted Kaplan–Meier (KM) estimates and Cox proportional hazards models providing hazard ratios (HRs) adjusted for the following baseline characteristics: age group (≤74, 75-84, ≥85 years), race (White, Black/African American, other/unknown), insurance coverage (commercial, public), index year for ARI initiation (2019-2020, 2021-2023), time (months) from nmCRPC diagnosis to index date, baseline PSA groups (<2, 2-<10, ≥10 ng/mL), baseline PSA doubling time (PSADT; ≤6, >6-≤10, >10 months, missing), and Gleason score at prostate cancer diagnosis (4-7, 8-10, missing). PSADT was calculated using the Sloan Kettering methodology,^26^ based on ≥3 PSA values ≥0.2 ng/mL taken ≥1 month apart within 12 months before the index date; PSADT values >100 months were capped at 100. State sequence plots were also generated for each treatment group,^18^ showing the proportion of patients in different disease states (receiving treatment, discontinuation, progression to mCRPC, lost to follow-up/death) at each time point during the study period.

Several sensitivity analyses were conducted to ensure robust findings. In some analyses, death was considered as a competing risk for discontinuation and mCRPC endpoints. In addition, a propensity score-based inverse probability of treatment weighting (IPTW) approach was used to minimize the influence of baseline confounding factors between treatment groups. Augmented IPTW was also performed. If there were any covariates with an SMD >0.2 after weighting, these were included in the IPTW model. It is a doubly robust estimator that uses propensity scores and outcomes models to balance out differences between groups and provides a more accurate estimate of the treatment effect.

In multivariate models, patients with missing baseline PSA or insurance information (approximately 96 patients) were assumed missing at random and excluded from the multivariate regression models. For variables with a substantial proportion of missing values (e.g., PSADT, Gleason score), a separate “missing” category was created and included in the model as a covariate. This approach assumed that patients with missing information shared sufficient similarities to be analyzed as a distinct group, although inclusion of this category also allowed for preservation of the overall sample size.

# Supplementary Results

### Sensitivity Analysis

Sensitivity analyses using IPTW to estimate the marginal HRs showed the same trend as the primary analysis results based on adjusted (conditional) HR. The IPTW HRs for ARI treatment discontinuation were: darolutamide vs enzalutamide HR 0.76 (95% confidence interval [CI] 0.63-0.91); darolutamide vs apalutamide HR 0.71 (95% CI 0.55-0.91); enzalutamide vs apalutamide HR 0.93 (95% CI 0.73-1.19). For mCRPC progression: darolutamide vs enzalutamide HR 0.65 (95% CI 0.52-0.82); darolutamide vs apalutamide HR 0.78 (95% CI 0.57-1.06); and enzalutamide vs apalutamide HR 1.20 (95% CI 0.89-1.60). For MFS: darolutamide vs enzalutamide HR 0.67 (95% CI 0.55-0.81); darolutamide vs apalutamide HR 0.75 (95% CI 0.58-0.98); enzalutamide vs apalutamide HR 1.13 (95% CI 0.88-1.46).

Consistent results were obtained when death was considered as a competing risk for discontinuation, and mCRPC endpoints. Similarly, the adjusted KM plots using IPTW, augmented IPTW, propensity score matching, or G-computation were consistent with the unadjusted KM plots regardless of the method we used to account for confounding factors. Adjusted KM plots using augmented IPTW are presented for the time to initial drug discontinuation (**Supplementary Figure 7**), time to mCRPC (**Supplementary Figure 8**), and time to MFS (**Supplementary Figure 9**) endpoints.

# Supplementary Figure 1. DEAR-EXT study design.

Abbreviations: ARI, androgen receptor inhibitor; EMR, electronic medical record; nmCRPC, nonmetastatic castration-resistant prostate cancer.

Figure previously presented at ASCO Quality Care Symposium 2024 (George DJ, et al. Poster presentation at ASCO Quality Care Symposium 2024: Abstract 399) and ASCO Genitourinary Symposium 2025 (George DJ, et al. Poster presentation at ASCO Genitourinary Symposium 2025: Abstract 158).

^a^Follow-up period: time from index date until date of last EMR activity, end of study period, or death, whichever occurred earliest.

^b^Patient identification and outcomes assessments based on EMR data provided by Precision Point Specialty Analytics, supplemented with information retrieved from patient charts.

^c^Unless the patient died sooner.

# Supplementary Figure 2. Patient flow.

Abbreviations: ARI, androgen receptor inhibitor; EMR, electronic medical record; NAH, novel
anti-hormonal; NMSC, non‑melanoma skin cancer; PPS, Precision Point Specialty Analytics.

# Supplementary Figure 3. Initial ARI starting year.

Patients initiating first ARI (%)

| Patients enrolled | Darolutamide (n = 565, 41%) | Enzalutamide (n = 609, 44%) | Apalutamide (n = 201, 15%) |
| --- | --- | --- | --- |
| 2019 | 23 | 57 | 19 |
| 2020 | 41 | 46 | 13 |
| 2021 | 45 | 41 | 15 |
| 2022 | 50 | 36 | 15 |
| 2023 | 44 | 47 | 9 |

Abbreviation: ARI, androgen receptor inhibitor.

# Supplementary Figure 4. Reasons for discontinuation of initial ARI.^a^

Abbreviations: AE, adverse event; ARI, androgen receptor inhibitor.

^a^Discontinuation defined as initial ARI treatment stop (and no restart of same treatment within 60 days), switch to another ARI, or death.

^b^Includes cost and drug–drug interaction.

# Supplementary Figure 5. HR estimates from adjusted Cox proportional hazards model for (A) time to initial ARI discontinuation,^a^ (B) time to progression to mCRPC, (C) MFS.

**(A)**

**(B)**

**(C)**

Abbreviations: ARI, androgen receptor inhibitor; CI, confidence interval; CRPC, castration-resistant prostate cancer; HR, hazard ratio; mCRPC, metastatic castration-resistant prostate cancer; MFS, metastasis-free survival; PSA, prostate-specific antigen; PSADT, prostate-specific antigen doubling time.

^a^Discontinuation defined as initial ARI treatment stop (and no restart of same treatment within 60 days), switch to another ARI, or death.

**Supplementary Figure 6.** Distribution of disease state by treatment group.

Abbreviations: APA, apalutamide; DARO, darolutamide; ENZA, enzalutamide; mCRPC, metastatic castration-resistant prostate cancer.

The percentages shown in the figure are examples of state distribution at 12 months.

**Supplementary Figure 7.** Augmented-IPTW adjusted Kaplan–Meier curves for time to initial drug discontinuation

# Abbreviations: ARI, androgen receptor inhibitor; IPTW, inverse probability of treatment weighting.

**Supplementary Figure 8.** Augmented-IPTW adjusted Kaplan–Meier curves for time to mCRPC

Abbreviations: ARI, androgen receptor inhibitor; IPTW, inverse probability of treatment weighting; mCRPC, metastatic castration-resistant prostate cancer.

# Supplementary Figure 9. Augmented-IPTW adjusted Kaplan–Meier curves for time to MFS.

Abbreviations: ARI, androgen receptor inhibitor; IPTW, inverse probability of treatment weighting;
MFS, metastasis-free survival.

**Supplementary Figure 10.** Covariate balance before and after implementation of IPTW adjustment for (A) darolutamide vs enzalutamide and (B) darolutamide vs apalutamide

Abbreviations: CRPC, castration-resistant prostate cancer; IPTW, inverse probability of treatment weighting; PSA, prostate-specific antigen; PSADT, prostate-specific antigen doubling time;
SMD, standardized mean difference.

# Supplementary Table 1. AEs Leading to Treatment Discontinuation^a^ (≥1% in any Treatment Group).

| **AE resulting in discontinuation** | **Darolutamide (n = 565)** | **Enzalutamide (n = 609)** | **Apalutamide  (n = 201)** | |
| --- | --- | --- | --- | --- |
| Any AE, n (%) | 54 (9.6) | 94 (15.4) | 30 (14.9) | |
| Fatigue | 24 (4.2) | 42 (6.9) | 8 (4.0) | |
| Pain | 13 (2.3) | 12 (2.0) | 2 (1.0) |  |
| Dizziness | 4 (0.7) | 12 (2.0) | 2 (1.0) | |
| Diarrhea | 6 (1.1) | 6 (1.0) | 5 (2.5) |  |
| Nausea | 5 (0.9) | 5 (0.8) | 4 (2.0) |  |
| Rash | 5 (0.9) | 0 | 7 (3.5) |  |
| Cognitive disorder | 3 (0.5) | 8 (1.3) | 3 (1.5) | |
| Fall | 4 (0.7) | 8 (1.3) | 0 | |

Abbreviation: AE, adverse event.

^a^Discontinuation defined as initial ARI treatment stop (and no restart of same treatment within 60 days), switch to another ARI, or death.

# Supplementary Table 2. Most Frequently Reported TEAEs of Interest.

| **TEAE** | **Darolutamide (n = 565)** | **Enzalutamide (n = 609)** | **Apalutamide (n = 201)** | |
| --- | --- | --- | --- | --- |
| Any AEs, n (%) | 142 (25.1) | 173 (28.4) | 60 (29.9) | |
| AE of interest,^a^ n (%) | 92 (16.3) | 103 (16.9) | 42 (20.9) | |
| Fatigue | 75 (13.3) | 81 (13.3) | 21 (10.4) |  |
| Rash | 13 (2.3) | 4 (0.7) | 15 (7.5) |  |
| Cognitive and memory mental impairment | 6 (1.1) | 10 (1.6) | 4 (2.0) |  |
| Fall | 4 (0.7) | 9 (1.5) | 1 (0.5) |  |
| Hypertension | 2 (0.4) | 6 (1.0) | 2 (1.0) |  |

Abbreviations: AE, adverse event; TEAE, treatment-emergent adverse event.

Table shows events reported by ≥1% of patients in any treatment group.

^a^AEs commonly associated with androgen receptor inhibitor therapy.
